# Right thyrocervical trunk rupture after right internal jugular vein puncture: a case report and systematic review of the literature

**DOI:** 10.1186/s40981-022-00565-w

**Published:** 2022-09-16

**Authors:** Yuko Ono, Eisuke Ueshima, Nobuto Nakanishi, Kazuaki Shinohara, Isamu Yamada, Joji Kotani

**Affiliations:** 1 Department of Disaster and Emergency Medicine, Graduate School of Medicine, Kobe University, 7-5-2 Kusunoki-cho, Chuo-ku, Kobe, Hyogo 650-0017 Japan; 2 Department of Anesthesiology, Ohta General Hospital Foundation, Ohta Nishinouchi Hospital, 2-5-20 Nishinouchi, Koriyama, Fukushima 963-8558 Japan; 3 Department of Diagnostic and Interventional Radiology, Kobe University, Kobe, Japan

**Keywords:** Accidental arterial puncture, Central venous catheter placement, Mechanical complication

## Abstract

**Background:**

Thyrocervical trunk rupture is an unusual, but critical, complication associated with central venous catheter (CVC) placement. The management of this complication has not been fully determined because it is rare.

**Case presentation:**

A 53-year-old Japanese woman with anorexia nervosa developed refractory ventricular fibrillation. After returning spontaneous circulation, a CVC was successfully placed at the initial attempt in the right internal jugular vein using real-time ultrasound guidance. Immediately after CVC placement, she developed enlarging swelling around the neck. Contrast-enhanced computed tomography showed massive contrast media extravasation around the neck and mediastinum. Brachiocephalic artery angiography showed a “blush” appearance of the ruptured right thyrocervical trunk. After selective arterial embolization with 33% N-butyl-2-cyanoacrylate, the extravasation completely disappeared and hemostasis was achieved.

**Conclusion:**

Our findings suggest that severe vascular complications arising from CVC placement can occur in patients with a fragile physiological state. Endovascular embolization is an effective treatment for such complications.

**Supplementary Information:**

The online version contains supplementary material available at 10.1186/s40981-022-00565-w.

## Background

Central venous catheter (CVC) placement is a common and crucial intervention for managing critically ill patients and one of the most essential skill competencies for anesthesiologists. The internal jugular vein (IJV) is the preferred site for CVC placement, but life-threatening mechanical complications can occur associated with IJV puncture [1, 2]. One of the most common mechanical complications associated with IJV cannulation is an accidental arterial puncture, mainly the carotid artery [3] because of its anatomical proximity to the IJV. Additionally, accidental arterial puncture can occur in the vertebral artery [4], innominate artery [5], internal mammary artery [6], and thyrocervical trunk [7–16]. Thyrocervical trunk rupture is an unusual, but life-threatening, complication associated with CVC placement. The detailed clinical situations, risk factors, and management of this complication have not been fully determined because it is rare [7–16]. Therefore, we report an illustrative case of this complication. We also conducted a comprehensive literature review of thyrocervical trunk injury after IJV puncture.

## Case presentation

A 53-year-old Japanese woman with a 30-year clinical history of anorexia nervosa (155 cm, 32 kg, body mass index: 13.3 kg/m^2^) was found unconscious at home. On admission to the Emergency Department, she was in a coma with a consciousness level of 6 on the Glasgow Coma Scale (E3V1M2). Her other vital signs initially recorded in the Emergency Department were as follows: body temperature, 35.1°C; heart rate, 83 beats/min; blood pressure, 84/62 mmHg; respiratory rate, 12 breaths/min; and percutaneous oxygen saturation, 98% (on oxygen 10 L/min via a non-rebreather mask). A laboratory examination showed remarkable hypoglycemia with a blood sugar concentration of 8 mg/dL, anemia with a hemoglobin concentration of 8.2 g/dL, hypopotassemia with a potassium concentration of 3.4 mmol/L, thrombocytopenia with a platelet count of 128,000/μL, and coagulopathy with an international normalized ratio of the prothrombin time of 1.49 and activated partial thromboplastin time of 36.2 s. After the intravenous administration of 20 g of glucose and 20 mg of thiamine, her blood sugar concentration increased to 230 mg/dL, and her consciousness level returned to 14 on the Glasgow Coma Scale (E3V5M6). Transthoracic echocardiography showed akinesis of the heart apex and a reduced left ventricular ejection fraction of 20%, consistent with takotsubo cardiomyopathy. She was obviously emaciated and malnourished but did not have a short neck or neck deformities. A computed tomography scan showed no vascular anomaly around the neck or thorax. A diagnosis of hypoglycemia-induced takotsubo cardiomyopathy [17, 18] with anorexia nervosa was made. She was admitted to the intensive care unit to receive close monitoring, correction of blood sugar and electrolytes, and continuous intravenous heparin administration (5000 U/day) for the prevention of thrombus in the akinetic ventricular apex.

**Fig. 1:**
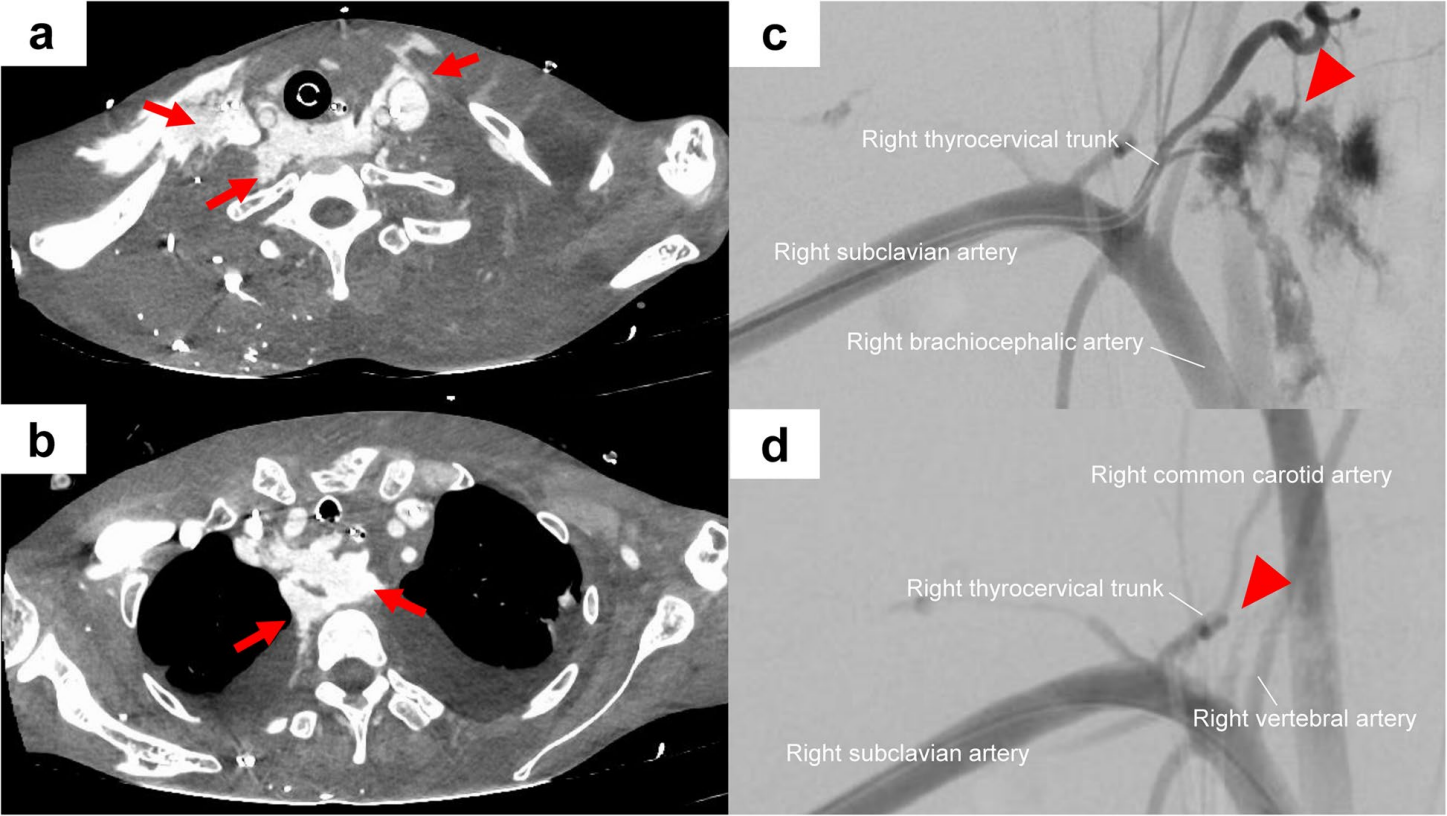
Massive bleeding from a ruptured right thyrocervical trunk associated with right internal jugular vein puncture. Contrast-enhanced computed tomography shows massive contrast media extravasation around the neck (**a**, arrows) and mediastinum (**b**, arrows). Successive brachiocephalic artery angiography shows a characteristic “blush” appearance of a ruptured right thyrocervical trunk (**c**, arrowhead). After selective arterial embolization using 33% N-butyl-2-cyanoacrylate, the extravasation has completely disappeared (**d**, arrowhead)

On hospital day 2, this patient suddenly developed refractory ventricular fibrillation and suffered from cardiopulmonary arrest. A rigorous resuscitation attempt using chest compressions, electrical defibrillation, endotracheal intubation, and intravenous adrenaline administration was immediately initiated by intensive care unit physicians and nurses. Spontaneous circulation was restored in approximately 15 min. A CVC was required for continuous intravenous infusion of inotropic agents and vasopressin. The right neck was then sterilized with 1% chlorhexidine digluconate, and her head was tilted to the left by approximately 30°. On a pre-procedural ultrasound (US) examination, no vascular-like structures were visible behind the posterior wall of the right IJV. The right IJV was punctured by an experienced cardiologist under US guidance with the short-axis out-of-plane technique using an 18-gauge introducer needle contained in an Arrow triple lumen central venous catheter kit (Teleflex Medical Japan, Tokyo, Japan). Although the angle and depth of the needle appeared to be appropriate if the patient had not been emaciated, the right IJV was completely collapsed at the time of the US-guided puncture, and blood was not aspirated. Non-pulsatile dark-colored blood was smoothly aspirated during gentle withdrawal of the needle, and then a guide wire, dilator, and CVC were inserted without resistance. Immediately after the CVC placement, she developed enlarging swelling around the neck. Contrast-enhanced computed tomography showed massive contrast media extravasation around the neck and mediastinum (Fig. 1a and b, respectively), whereas the tip of the CVC was correctly placed in the right IJV. The patient was considered at a high risk for open repair. Therefore, transarterial embolization was planned. After 6-Fr short sheath introduction (Radifocus Introducer II; Terumo, Tokyo, Japan) from her right brachial artery, a pigtail catheter (4-Fr; Cook Medical, Bloomington, IN, USA) was advanced into the brachiocephalic trunk with the support of a 0.035″ hydrophilic guidewire (Radifocus guidewire M; Terumo, Tokyo, Japan). Brachiocephalic artery angiography showed a characteristic “blush” appearance of the ruptured right thyrocervical trunk (Fig. 1c). After selective arterial embolization using a microcatheter (Masters Parkway Soft; Asahi Intecc, Aichi, Japan) and 33% N-butyl-2-cyanoacrylate, the extravasation completely disappeared (Fig. 1d) and hemostasis was achieved. A schema of the operative course at the catheterization laboratory is shown in Figure S1 in the supplementary information file. After receiving 22 units of packed red blood cells, 20 units of fresh frozen plasma, and 20 units of platelet concentrate, the patient was returned to the intensive care unit where correction of body fluid imbalance, hypothermia, acidemia, and coagulopathy was continued. Secondary infection and multiorgan dysfunction developed approximately 2 weeks later, and the patient died from sepsis on hospital day 16. An autopsy was not performed because her family refused.

### Systematic literature review

**Fig. 2:**
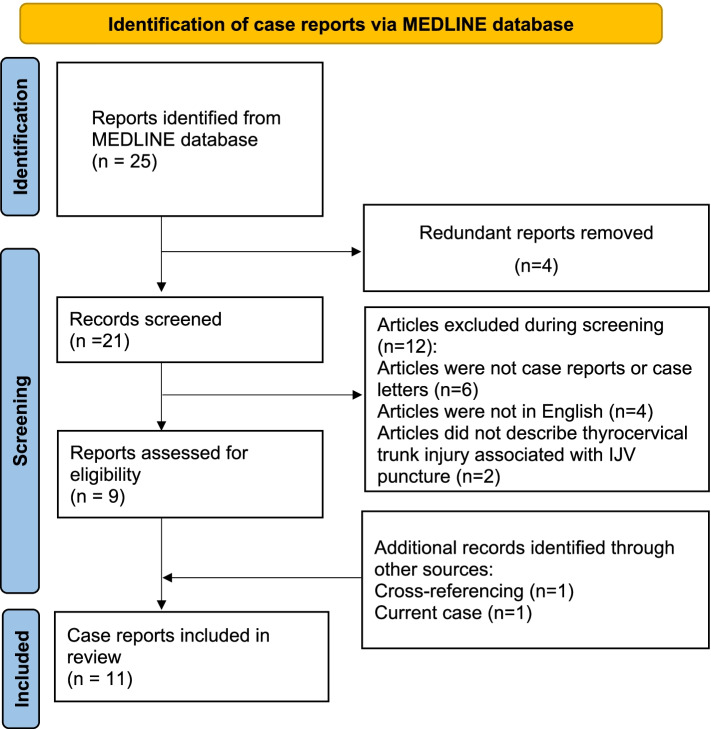
Flow diagram of the literature review. IJV, internal jugular vein

The search strategy was determined a priori by the survey team, which comprised anesthesiologists (YO and KS), an interventional radiologist (EU), emergency physicians (NN, IY, and JK), and a librarian (MJ, listed in the Acknowledgments). On June 2022, all reported cases of thyrocervical trunk injury associated with IJV puncture were searched for in the MEDLINE database from inception using the following keywords: “thyrocervical trunk” AND “internal jugular vein puncture”; “thyrocervical trunk” AND “central venous catheter”; “thyroid artery” AND “internal jugular vein puncture”; and “thyroid artery” AND “central venous catheter”. PubMed® (https://pubmed.ncbi.nlm.nih.gov/) was used to search the MEDLINE database. Cross-referencing was also performed using the reference list of articles included in this review. The following types of articles were excluded from the analysis: (1) they were not case reports or case letters, (2) they were not in English, and (3) they did not describe thyrocervical trunk injury associated with IJV puncture. This search produced 25 articles of which 10 relevant reports and cases were included in this review [7–16] (Fig. 2). We reviewed the variables of age, sex, underlying medical conditions of the patients, number of punctures, use of real-time US guidance, characteristics of the operator, treatment, and outcome.

The clinical characteristics of thyrocervical trunk injury after IJV puncture described in this review, including our patient, are shown in Table 1. Seven articles described pseudoaneurysm of the thyrocervical trunk or its branches [7–13], three reported massive bleeding arising from the thyrocervical trunk or its branches [14, 15], and one reported CVC misplacement in the right inferior thyroid artery [16]. The cases in the literature review consisted of four men and seven women, aged 33 to 71 years. More than 80% (9/11) of thyrocervical trunk injuries were associated with a landmark puncture without using real-time US guidance. More than half (6/11) of the thyrocervical trunk injuries were associated with multiple (≥ 2) attempts. Aneurysm or active bleeding arising from a thyrocervical trunk injury was successfully managed by surgical repair (4/11) or endovascular treatment (5/11). Endovascular embolization using 33% N-butyl-2-cyanoacrylate for the treatment of vascular complications associated with CVC placement has not been reported previously.

**Table 1:** Summary of the clinical characteristics of thyrocervical trunk injury after internal jugular vein puncture

Reference number	Details of mechanical complications	Age (years)/sex	Underlying medical condition	Number of attempts	Use of real-time US guidance	Characteristics of the operator	Treatment	Outcome
[7]	Right thyrocervical trunk pseudoaneurysm	33/female	Suspected pulmonary embolism after emergency cesarean section	1	Yes	NR	Endovascular repair with a balloon-expandable covered stent	Survival
[8]	Right thyrocervical trunk pseudoaneurysm	52/female	Acute on chronic renal failure	Several times	No	Nephrologist	Surgical ligation	Survival
[9]	Right thyrocervical trunk pseudoaneurysm	63/male	Recurrent sepsis syndrome of an unknown source	Several times	No	NR	Surgical ligation	Survival
[10]	Right thyrocervical trunk pseudoaneurysm	57/female	Renal failure due to autosomal dominant polycystic kidney disease	NR	No	Attending physician	Surgical ligation	Survival
[11]	Right inferior thyroid artery pseudoaneurysm	54/female	Severe esophagitis	Several times	No	NR	Endovascular coil embolization	Survival
[12]	Right inferior thyroid artery pseudoaneurysm	51/female	Elective right-sided hepatectomy because of hepatocellular carcinoma with liver cirrhosis	1	No	Third year resident	Endovascular coil embolization	Survival
[13]	Right transverse cervical artery pseudoaneurysm	46/male	End-stage renal failure	Several times	No	NR	Endovascular coil embolization	Survival
[14]	Right thyroid artery injury	49/male	Repair of an ascending aortic aneurysm	2	No	Attending anesthesiologist	Observation	Survival
[15]	Right superior thyroid artery rupture	44/female	End-stage renal failure	2	No	NR	Endovascular coil embolization	Survival
[16]	Central venous catheter misplaced in the right inferior thyroid artery	71/male	Left-sided hemi-hepatectomy because of central metastases of a rectal carcinoma	1	No	Attending anesthesiologist	Surgical repair	Survival
Current case	Right thyrocervical trunk rupture	53/female	Anorexia nervosa and takotsubo cardiomyopathy	1	Yes	Cardiologist	Endovascular embolization using 33% N-butyl-2-cyanoacrylate	Death

*NR* Not recorded, *US* Ultrasound

## Discussion

**Fig. 3:**
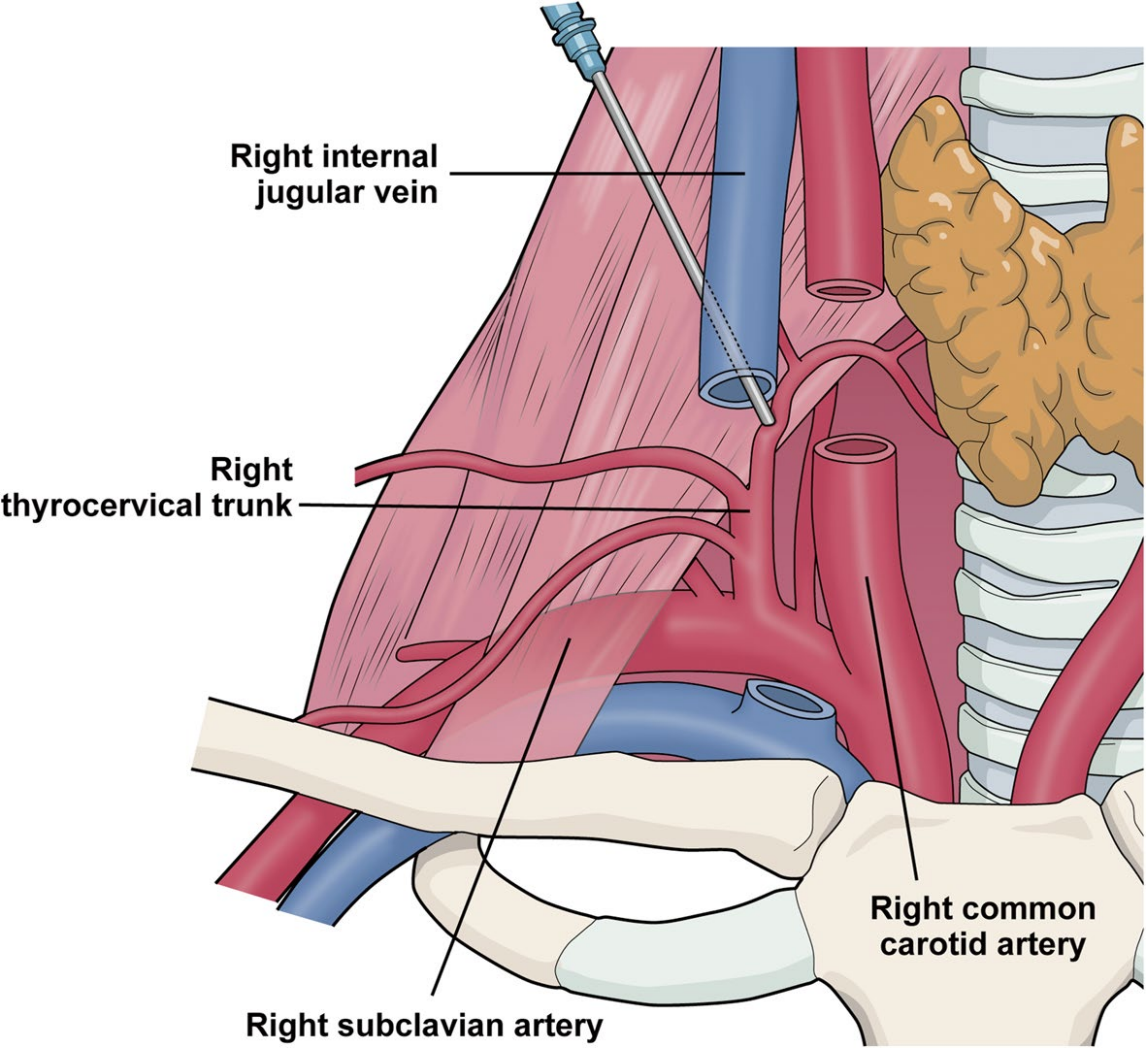
Schematic image of right thyrocervical trunk injury. During the placement of a central venous catheter, the introducer needle crossed the lumen of the right internal jugular vein, causing an accidental puncture of the thyrocervical trunk

This case involved severe complications arising from the right IJV puncture. In our case, real-time US-guided venipuncture was used by an experienced physician, and the CVC was successfully placed on the initial attempt. However, the introducer needle accidentally crossed the lumen of the IJV, with a subsequent puncture of the thyrocervical trunk (Fig. 3). The present case highlights the requirement for increased attention when attempting interventional vascular procedures in patients with anorexia nervosa. In our patient, bleeding from the ruptured thyrocervical trunk extended from the neck to the mediastinum through the prevertebral space, which is a large and loose compartment only bounded by deep cervical fascia. Transarterial embolization was helpful in the diagnosis and treatment of the thyrocervical trunk rupture associated with CVC placement.

In our literature review, more than 80% of thyrocervical trunk injuries were associated with a landmark puncture. Our review also showed that more than 50% of thyrocervical trunk injuries were associated with multiple (≥ 2) attempts. The current results are in agreement with the previous observations, which showed that the complication rate associated with CVC placement increased when more than two punctures were required [19, 20]. Previous randomized, controlled trials [21, 22] indicated that real-time US-guided CVC placement in the IJV had a higher success rate of a first insertion attempt and a lower rate of arterial puncture than the landmark-guided technique. Our results, together with these previous findings [19–22], collectively support the strategy of limiting the number of puncture attempts by using real-time US guidance.

In this case, real-time US-guided venipuncture was used by a skilled physician, and a CVC was successfully placed in the IJV at the initial attempt. However, right thyrocervical trunk rupture occurred and was associated with CVC placement. There are several plausible factors that might have caused this complication. First, this patient was obviously emaciated (body mass index: 13.3 kg/m^2^) and malnourished. The skin, connective tissue, and vascular walls were thin and fragile, which may have made the introducer needle cross the lumen of the IJV. A previous study also showed that a low body mass index (< 20 kg/m^2^) was a risk factor of mechanical complications associated with CVC placement [23]. Second, patients with anorexia nervosa are likely to have vascular abnormalities [24]. In response to starvation and energy deprivation, the connective tissue of the vascular wall is considerably weakened, making the vascular structures vulnerable. Launay et al. also described the case of an 18-year-old woman with anorexia nervosa who developed radial artery injury leading to digital necrosis following a radial arterial puncture for blood gas analysis [25]. Their findings together with our experience suggest that health care providers should carefully consider the need for invasive vascular procedures in this tenuous patient population. Third, in addition to underlying thrombocytopenia and coagulopathy, our patient received continuous intravenous heparin administration for the prevention of thrombus in the akinetic ventricular apex. Although the abnormal coagulation profile was not the main cause of bleeding in this case, it could have been an aggravating factor in the rapid progression of the neck hematoma. This possibility indicates that blood coagulation function should be determined before attempting to cannulate the IJV.

In this case, the right IJV collapsed during real-time US-guided puncture, and the thick introducer needle (18 gauge) was unintendedly deeply advanced. In such a situation, inadvertent artery cannulation may develop. Previous reports have shown that CVC misplacement in the carotid artery [26], vertebral artery [27], subclavian artery [28], and thyrocervical trunk [16] can occur associated with IJV puncture, regardless of the use of the US-guided technique. The management of the consequences of arterial placement of a large-bore catheter is challenging. Once the catheter is removed, direct manual pressure may be difficult to place on the artery because it is usually distal to the puncture site in the skin [16]. In fact, pulling the catheter out and applying manual pressure is associated with severe complications, such as persistent bleeding, hematoma, arteriovenous fistula, pseudoaneurysm, stroke, and death [2]. Therefore, a vascular surgeon should be consulted before removing the misplaced catheter [16]. The surgeon should decide whether to explore the vessel and what type of repair may be required.

Finally, this case illustrates the successful management of the thyrocervical trunk by endovascular treatment. Carotid angiography was helpful in the diagnosis and treatment of bleeding. After selective arterial embolization using 33% N-butyl-2-cyanoacrylate, extravasation completely disappeared and hemostasis was achieved. Our literature review also showed that more than half of the cases of aneurysms and intractable hemorrhage arising from thyroid artery injury were successfully managed by endovascular treatment. Although surgical exploration was another therapeutic option for controlling the hemorrhage, open repair was not considered appropriate in our patient because she was at a high risk of intraoperative bleeding and anastomotic leakage for concomitant comorbidities. Based on these observations, endovascular therapy appears to be a rapid and effective option for treating vascular accidents associated with CVC placement, especially in those with a fragile physiological status.

In conclusion, we report a right thyrocervical trunk rupture after a right IJV puncture in a Japanese woman with anorexia nervosa. The findings in this case suggest that severe mechanical complications arising from CVC placement can occur, especially in patients with a fragile physiological state. Our findings also suggest that endovascular embolization is an effective treatment for such complications. This case indicates the need for caution in all health care professionals who are involved in this common and important intervention.

## Supplementary Information

**Additional file 1: Fig. S1.** Schema of the operative course at the catheterization laboratory.

## Data Availability

The datasets used during the literature review are available from the corresponding author upon reasonable request.

## Abbreviations

CVC: Central venous catheter
IJV: Internal jugular vein
US: Ultrasound
