# Supplementary material for: Right thyrocervical trunk rupture after right internal jugular vein puncture: a case report and systematic review of the literature
Source: JA Clin Rep. 2022 Sep 16;8:74. doi: 10.1186/s40981-022-00565-w (PMC9477995; doi:10.1186/s40981-022-00565-w)
Supplement: Supplementary file 1 — Additional file 1: Fig. S1. Schema of the operative course at the catheterization laboratory. [file 40981_2022_565_MOESM1_ESM.pdf]

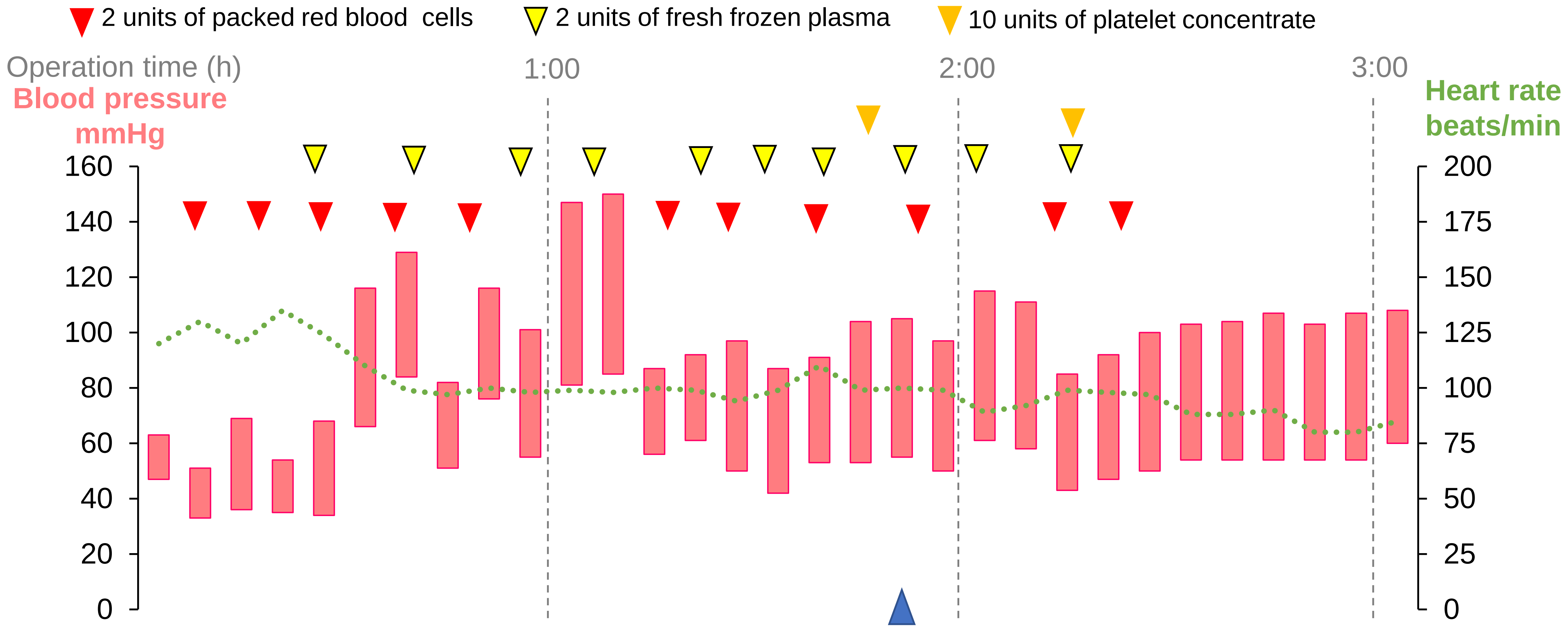

Hemoglobin (g/dL)	7.1	3.5	6.9
Platelets (x10[4]/μL)	9.0	1.4	3.1
Fibrinogen (mg/dL)	114	58	128
APTT (sec)	110	>180	63.6
PT-INR	2.10	3.10	2.10

Figure S1. Schema of the operative course at the catheterization laboratory.
PT-INR: international normalized ratio of the prothrombin time; APTT: activated partial thromboplastin time.
